# Evidence for self-sustaining populations of *Arcuatula senhousia* in the UK and a review of this species’ potential impacts within Europe

**DOI:** 10.1038/s41598-021-86876-x

**Published:** 2021-05-06

**Authors:** Gordon James Watson, Jesie Dyos, Peter Barfield, Paul Stebbing, Kate Gabrielle Dey

**Affiliations:** 1 Institute of Marine Sciences, School of Biological Sciences, University of Portsmouth, Ferry Road, Portsmouth, PO4 9LY UK; 2 APEM Ltd, A17 Embankment Business Park, Heaton Mersey, Manchester, SK4 3GN UK

**Keywords:** Invasive species, Conservation biology, Population dynamics

## Abstract

The invasive Asian date mussel (*Arcuatula senhousia*) inhabits diverse global coastal environments, in some circumstances posing significant ecological and economic risks. Recently recorded in the Greater North Sea ecoregion, an established population has not previously been confirmed. Combining historical and field data, we provided baseline information from the UK and recorded colonisation in a variety of habitats. Gonadal development was assessed using the gonadosomatic index (GSI) to determine if an intertidal soft-sediment population is self-sustaining. *Arcuatula senhousia* records from subtidal muddy/mixed-sediment within a major estuarine system from 2007 to 2016 were also analysed. First detected in 2011*,* spatial distribution was variable across the years within the subtidal, with individuals found at 4–9 out of 25 sites, and densities per site varying from 10 to 290 individuals per m^2^. The intertidal population was, in part, associated with seagrass (*Zostera* spp.) and attached to bivalves. In marinas, individuals were attached to concrete tiles, associated with live *Mytilus edulis*, and to dead *Ostrea edulis*. Mean GSI from the intertidal population differed across months, peaking in July before declining in September/October, but with high inter-individual variability. *Arcuatula senhousia* is reproducing and maintaining viable populations. Using a natural capital approach, we identify the potential impacts on Europe’s functionally important habitats, fisheries and aquaculture if its spread continues.

## Introduction

*Arcuatula senhousia* (Benson, 1842), formerly known as *Musculista senhousia,* and commonly known as the Asian date mussel, is a fast-growing, relatively small (< 40 mm in length), mytilid mussel which can be found in intertidal and subtidal habitats^1,2^. Its vast native range stretching from Singapore to Siberia^3,4^ is testament to its environmental adaptability which has led to its extensive distribution as a non-native species^5^. As a non-native, it was first detected on the Pacific coast of North America in the 1920s^6^, but has also been reported from Australia and New Zealand^7,8^; the Mediterranean and Adriatic Seas^9–12^; the Azov-Black Sea Basin^13^ and West Africa^14^. Finally, it has been reported from the Suez Canal, Red Sea, Aden, Zanzibar, Madagascar, Mauritius, India, Indo-China and New Caledonia^15,16^.

Successful *A. senshousia* introductions have been attributed to traits typical of invasive species: high fecundity; high dispersal capability; fast growth rate; phenotypic plasticity and tolerance to a wide range of environmental conditions^1,3,17,18^. Although small in size, a female can release > 100,000 eggs^19^, preceding an extended larval planktonic stage lasting two to eight weeks facilitating dispersal^16,20,21^. Once settled, individuals can mature within nine months^22^ adapting their reproductive cycle to new conditions^1,19,23^.

In Europe, *A. senhousia* was reported from Arcachon Bay, on the Atlantic coast of France in 2002^24^. There were no further reports north of this location until 2017, when *A. senhousia* was detected in the Solent region of the south coast of England using eDNA metabarcoding and five specimens were found on intertidal sediment within the same region^25,26^. However, the distribution and abundance of the species in the Solent are not known. The Solent is a 32 km long strait that separates the Isle of Wight from mainland England that sits within the ecoregion of the Greater North Sea (this ecoregion encompasses the coastlines of the UK, France, Belgium, Netherlands, Germany, Denmark, Sweden and Norway^27^). The Solent hosts a diverse range of temperate coastal habitats, with annual sea surface temperatures typically varying from 9 °C (February) to 17 °C (September)^28^. It is protected under a variety of local, national and international conservation designations due to important habitats and the biodiversity they support^29^. The Solent is also subject to great anthropogenic pressure, for example hosting international commercial ports and high levels of recreational boating activity.

Invasive species alter the value of ecosystems in terms of the benefits that people obtain from them (ecosystem goods and services)^30^. Ecosystem engineers, such as *A. senhousia,* at high population densities, can be particularly influential due to their ability to modify, create or destroy habitat^31^. In doing so they impact natural capital, here defined as “…the stock of forests, rivers, land, minerals and oceans, as well as the natural processes and functions that underpin their operation”^32^. Impacts of non-native species on commercially and ecologically important species as well as native habitats are poorly understood^33^ and this is also the case for *A. senhousia*. In the North Pacific there is evidence for *A. senhousia* inhibiting rhizome growth of seagrass (*Zostera marina*) where *Z. marina* is patchy and sparse^34^, however interactions between the two species are complex; for instance *A. senhousia* fertilise *Z. marina* beds^35,36^. *A. senhousia* can also attach to hard surfaces and so has the potential to foul and outcompete cultured bivalves^37^. As an autogenic ecosystem engineer (conspecifics can bind together using byssal threads to form dense mats in its native and non-native ranges^22,38,39^) high numbers not only alter the space and type of available substrate but also sediment conditions^40,41^. Intertidal soft sediments, seagrasses (*Zostera* spp.) and the ecologically and commercially important European flat oyster (*Ostrea edulis*) may all be affected. An established Solent population could also affect commercially important shellfish, and bait fisheries that extract significant biomass from soft-sediment benthic habitats^42,43^.

Evaluation of risk associated with a non-native species is based on many factors including introduction, establishment and spread potential as well as impacts. This study provides a first step towards the assessment of risk associated with *A. senhousia* in European waters. Our aims are to: (i) Assess spatial distribution and temporal trends in a subtidal population from Southampton Water, using historic data from routine coastal surveys performed by the Environment Agency (EA); (ii) Provide the first baseline information on the species’ presence within the Solent and confirm its ability to colonise diverse habitats within the Greater North Sea, using a combination of the historic data and our own field data (2019); (iii) Investigate if an intertidal sediment population of the Solent has the potential to deliver larvae across this region by assessing gonadal development and gametogenic processes; (iv) Identify potential effects (positive and negative) of *A. senhousia* for species, habitats, fisheries and aquaculture (i.e. goods and services) of Europe if it spreads beyond the Solent region, with reference to current literature and data from this study.

## Methods

### Assessment of spatial distribution and temporal trends

Subtidal surveys in Southampton Water were undertaken from 2007 to 2016 when the EA carried out routine benthic surveys as part of the monitoring programme for the UK government’s Water Framework Directive (WFD)^44^. Forty-five sites (2007) and 25 sites (2011, 2013 and 2016) within Southampton Water and its estuaries (Rivers Test, Itchen and Hamble) were semi-randomly selected for sampling each year by considering sediment type, accessibility and potential hazards. A site was approximately defined as a 50 m radius surrounding a target coordinate. One grab sample, using a 0.1 m^2^ Day grab, was taken to assess macrofauna at each site. Macrofauna processing and identification were undertaken by a contractor using standard operating and quality control procedures used by the industry (e.g. NMBAQCS: North East Atlantic Marine Biological Analytical Quality Control Scheme) with macrofauna extracted using a 0.5 mm sieve. No specific size measurements of *A. senhousia* were recorded.

### Assessment of spatial distribution, gonad staging, habitat preference

The intertidal shore at Brownwich was surveyed in 2019 using six 600 m × 5 m transects parallel to the mean low water springtide line, evenly spaced (by 40 m) from high shore to low shore. The surveyor walked within the transect parameters locating *A. senhousia* that were immediately apparent on the sediment surface without sediment excavation. *Arcuatula senhousia* locations were recorded using a GPS device (Garmin eTrex 20x) and shell lengths measured using calipers. Every other measured specimen was transported back to the laboratory and fixed in formalin before the gonadosomatic index (GSI) measurements were obtained. For GSI, gonads and other tissue were dissected and then calculated as follows: ([gonad wet weight (g) / bodyweight without shell (g)] × 100)^45^.

Surveys not targeted at detecting *A. senhousia* also provided records for this species from intertidal locations within the Solent region. These surveys were conducted by researchers from the Universities of Portsmouth and Southampton, a volunteer for the Hampshire and Isle of Wight Wildlife Trust and Pisces Conservation Ltd (see Supplementary Table S1). From west to east, surveys included an intertidal macrobenthos survey at Lepe (2019), a fish push-net survey within the River Test (2016) and a seagrass quadrat survey at Portsmouth Harbour (2019). A specimen from the River Itchen (2018) was also found during an intentional search for *A. senhousia* on mudflats (no methodology recorded). Survey details regarding the specimen found at Chichester Harbour (2019) cannot be provided due to the commercial sensitivity of the location where it was found.

### Marina and harbour surveys across the Solent

As part of the Solent Oyster Restoration Project^46^, *O. edulis* were purchased in 2016 from the commissioned dredge fishery in Langstone Harbour and were translocated from the seabed into broodstock cages deployed at various locations within the Solent including Saxon Wharf (River Itchen). It should be noted that oysters were not cleaned of epifauna before translocation, in part due to the sheer numbers of oysters being moved. In total, approximately 10,000 *O. edulis* were purchased from the fishery, with each oyster being at least 3 years in age (> 70 mm). The oysters remained in the cages throughout 2017 and 2018 until the trial concluded in November 2018. At the end of the trial deceased individuals were extracted from the cages and taken to the laboratory where any *A. senhousia* which had colonised the shells were removed and shell lengths recorded using calipers. As part of the same restoration project, cages containing *O. edulis* were deployed in 2019 for nine months at Port Hamble in the River Hamble. *Arcuatula senhousia* individuals were found within the cages during scheduled monitoring of the oysters in April 2019, at which point they were collected, and shell size recorded. Macrobenthos samples were collected from subtidal sites around the Solent in 2019 to investigate possible associations between *O. edulis* and other macrobenthos. In addition, roof tiles (Burton Roofing Merchants Limited, Redland Plain Tile Antique Red, concrete: 27.0 × 16.5 × 1.0 cm) submerged at a depth of between 0.5–1.0 m for six months on pontoons in Saxon Wharf Marina, originally deployed to assess *O. edulis* settlement, were removed and placed in flow-through laboratory holding tanks for 21 days prior to the removal of all epifauna.

### Statistical analysis

A Kruskal–Wallis test (SPSS v.25) was used to determine whether there was a significant difference between median densities per site of *A. senhousia* individuals collected from each of the three EA surveys when *A. senhousia* was detected (2011, 2013 and 2016). This test was chosen because data were not normal and, due to the high number of zeros, could not be transformed. This test was also used to identify significant differences between the median GSI reported for March, May, July and September/October (data were collected during the last week of September and the first three weeks of October and were, therefore, combined). In order to identify which months had a significantly different GSI, pairwise-comparisons were subsequently made using the Wilcoxon rank sum test.

### Assessment of potential impacts

A literature review was conducted to gather information on *A. senhousia* impacts, specifically in relation to natural capital and vulnerable and protected habitats and species. To extract the relevant information, Web of Science and Google Scholar were used to search for common names and synonyms for *A. senhousia* as listed by CABI^5^. Other key words searched included “*Zostera*”, “impact”, “distribution”, “competition”, “clam”, “oyster” and “reproduction”. Impacts were then categorised by the relevant ecosystem services using the commonly used top level categories of Provisioning, Regulating, Cultural and Supporting e.g.^47,48^. We adapted these definitions to be the following: Provisioning services are products that people obtain from ecosystems (e.g. food and other raw materials); Regulating services are benefits that people obtain from the regulation of ecosystem processes (e.g. climate regulation and water purification); Cultural services are the non-material benefits that people obtain from ecosystems (e.g. recreation and health); Supporting services are those that are necessary for the production of all other ecosystem services (e.g. habitat provision and genetic diversity).

## Results

### Spatial distribution, temporal trends, habitat preference

**Figure 1:**
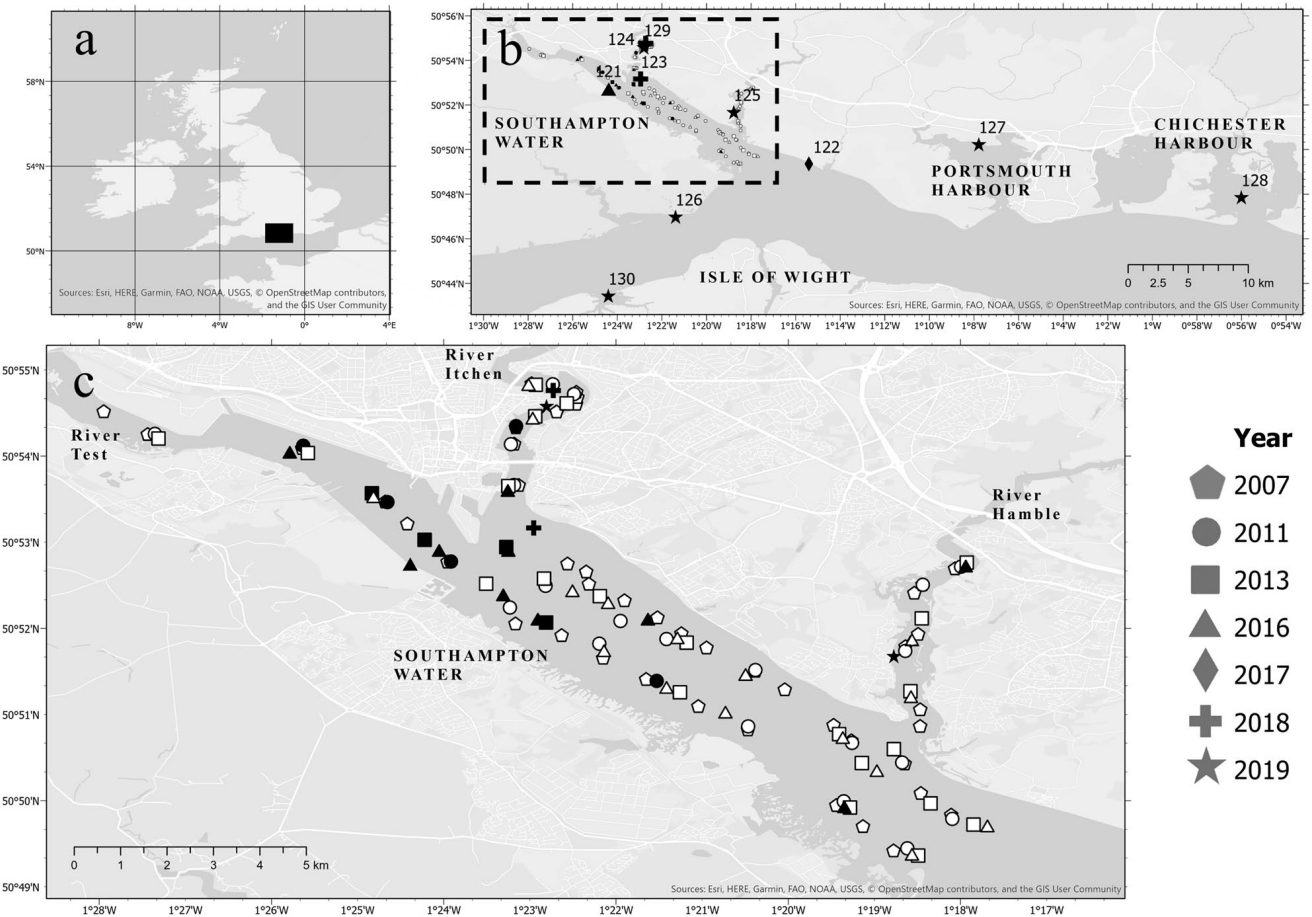
(**a**) Map of the UK denoting the Solent survey region. (**b**) Locations across the Solent region where *A. senhousia* has been detected. (**c**) Distribution of *A. senhousia* in the Solent focusing on Southampton Water and its tributaries as determined by EA benthic surveys. Dashed rectangle in (**b**) denotes area (**c**). Black fill indicates presence of *A. senhousia*, white fill indicates absence. Overlapping symbols are layered in order of year (most recent at the top). Symbols *without* numbers are Environment Agency (EA) survey sites (site numbers excluded to maximise clarity of map); EA site locations and associated *A. senhousia* densities can be found in Supplementary Table S2. Numbers 121–130 refer to surveys by other organisations (see Table 1). Mean densities for all surveys can be seen in Table 1. Site 128 is not a specific location but represents one individual found in Chichester harbour. Map created using ArcGIS Pro 2.6 https://pro.arcgis.com/. The intertidal shore at Brownwich (Fig. 1; site 122) was comprehensively surveyed in 2019. Compared to the subtidal sites in Southampton Water, the population density was low, with only 169 individuals recorded equivalent to 0.06 m^−2^ (Table 1). Single individuals were found mainly on the higher part of the shore partially buried in the sediment. None were attached to seagrass (*Zostera* spp.), however, when removed from the sediment a number were attached by their byssal threads to dead cockles (*Cerastoderma edule*) (empty shells) and living individuals. *Arcuatula senhousia* shell lengths ranged from 9 to 32 mm (mean = 20.1 + /− 3.9 SD).

The first scientifically reported sighting of *A. senhousia* in the UK prior to this study was from 2017^26^, however our study confirms the presence of this species in the UK since 2011 (mean *A. senhousia* densities for each survey can be found in Table 1). Routine surveys undertaken by the EA throughout Southampton Water and its three estuaries recorded the presence of *A. senhousia* from 2011–2016. In 2007, no *A. senhousia* individuals were found at any of the 45 sites (Fig. 1; sites 1–45, Supplementary Table S2). In 2011, five out of the 25 sites sampled contained *A. senhousia*, concentrated towards the upper reaches of the estuarine system (Fig. 1), and densities varied from 0 to 70 individuals per m^2^ (m^−2^) (mean = 7.2 + /− 18.6 SD) (sites 46–70, Supplementary Table S2). In 2013, samples from four out of the 25 sites contained *A. senhousia* (Fig. 1) with densities ranging from 0 to 70 m^−2^ (mean = 4.0 + /− 14.1 SD) (sites 71–95 in Supplementary Table S2). In 2016, *A. senhousia* was found at more sites (nine out of 25) across a greater geographic area (Fig. 1). For example, it was detected for the first time in the River Hamble and near the mouth of Southampton Water. The highest density was recorded in 2016 when there was a range of 0–290 m^−2^ (mean = 20.4 + /−58.8 SD) (sites 96–120 in Supplementary Table S2). Nevertheless, there is no significant difference in *A. senhousia* median density per site between 2011, 2013 and 2016 (Kruskal–Wallis test, X^2^(2) = 3.1, p = 0.215).

**Table 1:** Summary of *A. senhousia* population data from sites within the Solent region of the UK, recorded from 2007–2019. Site numbers correlate with Fig. 1. Gonad stages based on those of Sgro et al.^19^: “1–2” = spent or developing; “3–4” = ripe or spawning; “–” = data not collected.

Habitat	Location	Site	Year	Count	Density (m^−2^): range, mean, + /− SD	Shell length (mm): range, mean, + /− SD	Gonad stage	Surveying organisation
Subtidal	Southampton Water	1–45	2007	0	–	–	–	Environment Agency
		46–70	2011	18	0–70, 7.2 + /− 18.6	–	–	
		71–95	2013	10	0–70, 4 + /− 14.1	–	–	
		96–120	2016	51	0–290, 20.4 + /− 58.8	–	–	
Intertidal	Hythe, River Test	121	2016	1	–	17	–	Pisces Conservation Ltd
			2019	1	–	18	–	
Intertidal	Brownwich	122	2017	5	–	14.1–20.8, 17.9 + /− 2.5	–	University of Portsmouth
			2019	169	0.06	9–32, 20.1 + /− 3.9	March–May: 1–2; July: 3–4; Sept/Oct: 1–4 (in 2019)	
Intertidal	Weston Shore, River Itchen	123	2018	2	–	–	–	University of Southampton
Marina; suspended hard surfaces	Saxon Wharf, River Itchen	124	2018	3	0.67	7–13, 8.7 + /− 3.1	–	University of Portsmouth
				14	–	13–23, 17.6 + /− 3.0	–	
Marina; suspended hard surfaces	Port Hamble, River Hamble	125	2019	–	–	–	–	University of Portsmouth
Intertidal	Lepe	126	2019	1	–	–	–	Hampshire and Isle of Wight Wildlife Trust volunteer
Intertidal; *Zostera marina*, *Z. noltei* beds	Portsmouth Harbour	127	2019	1	4	18	–	University of Portsmouth
Intertidal; highly sheltered	Chichester Harbour	128	2019	1	–	4	–	University of Portsmouth
Marina; suspended hard surfaces	Shamrock Quay, River Itchen	129	2019	2	–	19, 28	–	University of Portsmouth
Subtidal	Newtown, Isle of Wight	130	2019	1	–	21	–	University of Portsmouth

In addition to the EA’s surveys, there have been further reports of *A. senhousia* from a variety of intertidal and marina surveys in all three rivers which discharge into Southampton Water (see Table 1 for survey details and site numbers). Two individuals were found near Hythe at the mouth of the River Test (Fig. 1; site 121), one in 2016 (17 mm length) and another in 2019 (18 mm length). In 2018, two individuals were recorded from Weston Shore in the River Itchen (Fig. 1; site 123). Further, *A. senhousia* were found attached to empty adult shells of *O. edulis* that had been removed from oyster cages at Saxon Wharf (Fig. 1; site 124), also in the River Itchen. Fourteen *A. senhousia* individuals ranging from 13–23 mm (mean = 17.6 + /− 3.0 SD) were removed from the oysters. Concrete tiles deployed in Saxon Wharf Marina (Fig. 1; site 124) had three individuals (mean = 8.7 + /− 3.1 SD) attached to the tiles or to *Mytilus edulis* when recovered in 2019. Two individuals (19 mm and 28 mm in length) were also found at Shamrock Quay (2019) attached to the metal cages housing the oysters (Fig. 1; site 129). An unknown number of *A. senhousia* individuals were also collected from oyster cages at Port Hamble (River Hamble). They were found attached to cockles and *Ulva* spp. that had been caught in cages suspended beneath the marina pontoons (Fig. 1; site 125).

Reports from three intertidal surveys and one subtidal survey provide evidence for the conclusion that *A. senhousia* is distributed across the Solent region. In 2019, one individual was found in Lepe in the west of the Solent (Fig. 1; site 126). To the east, one individual (18 mm length) was recovered from Portsmouth Harbour (Fig. 1; site 127) growing on mixed eelgrass (*Zostera marina* and *Z. noltei*) alongside significant quantities of *Ruppia* spp. Another individual (4 mm in length) was found on muddy sediment in highly sheltered conditions within Chichester Harbour (Fig. 1; site 128, but exact location cannot be disclosed due to commercial sensitivity of the survey) and another was recovered from the Isle of Wight in Newtown (Fig. 1; site 130).

### Gonad staging

Ninety-four individuals collected from Brownwich between March and September/October in 2019 were assessed for reproductive state. Gonad tissue lining the shells of *A. senhousia* collected in March ranged from extremely thin and barely visible (Supplementary Fig. S1a) to thin translucent tissue with white venation (Supplementary Fig. S1b). The translucent tissue corresponds to a high volume of follicle cells with collapsed or empty gametes indicating spent or developing gonads with no clear differences between sexes^19^. *Arcuatula senhousia* from May also resembled those collected in March, however, by July gonad tissue had substantially thickened and channels within the tissue could be seen (Supplementary Fig. S1c, d), suggesting that the gonads were ripe or at the spawning stage^19^. The colour of the gonads, either white (male) (Supplementary Fig. S1c) or orange (female) (Supplementary Fig. S1d) was also discernible confirming a 3F:2M sex ratio for the 15 *A. senhousia* individuals collected. By September/October there was a high inter-individual variation in reproductive state, with gonad stage appearing to range from spent to ripe/spawning. One out of the 12 individuals collected in September/October was identified as a female, although a sex ratio could not be established due to the thin gonad tissue of many of the mussels.

**Figure 2:**
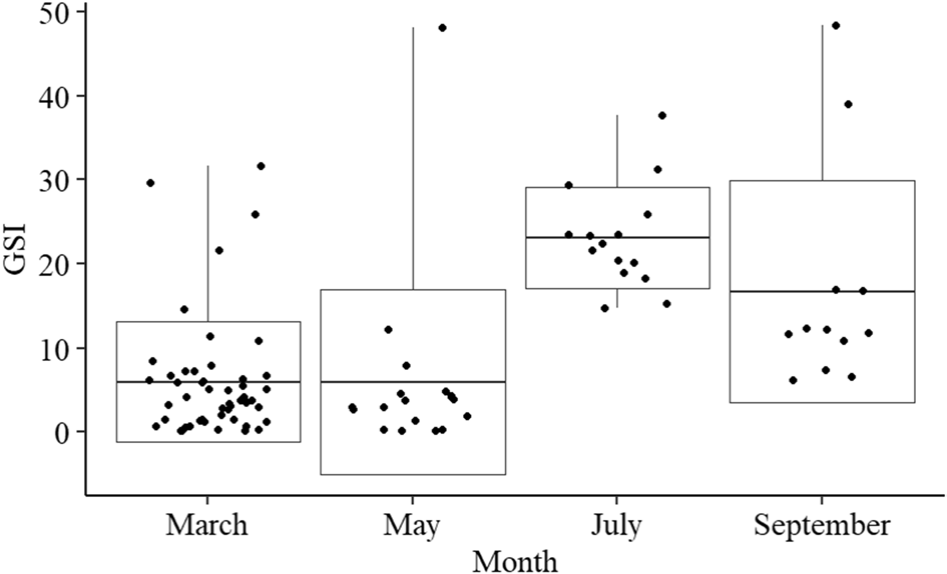
GSI for *A. senhousia* collected in March (n = 49), May (n = 18), July (n = 15) and September/October (n = 12) in 2019. The mean values are represented by the line in the centre of the box. Upper and lower limits of the box represent one standard deviation (SD). The whiskers represent data outside of one SD from the mean. Individual GSI data points are represented by the black dots.

To support the gross anatomical observations the GSI was calculated for each month and presented in Fig. 2. Mean GSI was low for both March and May (6.0 + /− 7.2 SD, 5.9 + /− 11.0 SD, respectively), but had increased to 23.1 + /− 6.1 SD by July. By September/October the mean GSI had decreased but remained higher than for March and May (16.7 + /− 13.3 SD). A Kruskal–Wallis test confirms that there are significant differences in median GSIs between the months sampled (Kruskal–Wallis, X^2^(3) = 41.5, p =  < 0.001). A pairwise-comparison of the median GSI for each month indicates that all months are significantly different from each other (Wilcoxon rank sum test, p =  < 0.05) apart from March and May.

## Discussion

### Baseline biological data and spatial distribution

Our data suggests that *A. senhousia* arrived in the UK, between 2007 and 2011, which was recently confirmed by Worsfold et al.^49^. The closest (in distance) European record of *A. senhousia* prior to this was from Arcachon Bay (Bay of Biscay), on the Atlantic coast of France in 2002^24^. The lack of reported sightings between the UK and the Bay of Biscay suggests a direct introduction event in the Solent as opposed to natural dispersal. As stated by Barfield et al.^26^, if the French population had gradually extended northwards unaided by any direct anthropogenic vector, it is reasonable to assume that its presence would have been recorded elsewhere before it reached the UK. However, spread of *A. senhousia* towards UK could have gone undetected due to limited monitoring for non-native species in the region. Potential vectors for introduction to the Solent include as a hitch-hiker with aquaculture species/produce^50^, but introduction by shipping is most likely. This is supported by the species’ ability to foul boat hulls^51^ and the detection of *A. senhousia* DNA in ballast water of boats in Dutch harbours^52^. A phylogenetic analysis is required to fully explore the likely invasion route(s) into the Solent and contextualise the global colonisation process. Attachment to seaweeds such as *Ulva* spp., as found in this study, could facilitate more local spread of *A. senhousia* by acting as a raft for hitchhikers (e.g.^53^).

Individuals collected ranged in size from 4 mm (Chichester Harbour) to 32 mm (Brownwich shore). Whilst Huber^2^ indicates an upper length of 40 mm for this species, an upper size limit of around 30–35 mm in its non-native range is most common in the literature (e.g.^1,8,24,54^). Linked to the small size in terms of traits of a successful invader is the short lifespan with most individuals living for only a year. Morton^17^ concluded that the small fraction of the population that lives up to two years is an adaptation for the continued survival of population in a variable environment. Considering a growth rate of approximately 2 mm a month depending on environmental conditions ^1,16,55^ it is possible that a few individuals at Brownwich were potentially older than a year. The size ranges recorded here, combined with the fact that individuals have been recorded from three sites on multiple years (Southampton Water: 2011, 2013, 2016; River Test: 2016, 2019; Brownwich: 2017, 2019) strongly suggest multiple generations from established populations.

Any self-sustaining population requires successful reproduction. While this is supported by the size ranges of *A. senhousia* (which spanned the 14–20 mm length maturity threshold^19,23^) the strongest evidence comes from the gonad imagery and GSI scores. From March to May gonads from individuals were not developed, but by July the significant increase in GSI combined with the typical gross morphology for bivalves confirms that individuals have maturing/mature gonads. By September/October, observation of the gonad tissue and the decrease in GSI suggest that some may have spawned. However, timings of the reproductive cycle need to be confirmed by histological analyses and plankton trawls.

The timings of these reproductive stages likely coincides with changes in water temperature; a variable which is well-documented for influencing bivalve reproduction and development^56,57^, especially in temperate regions^58^. In its native range of the Sea of Okhotsk, Southern Sakhalin (Russia), the spawning period of *A. senhousia* coincides with temperatures of 15–20°C^3^. This temperature range matches the inshore summer temperatures of the Solent (Watson, unpublished data) suggesting summer spawning in Europe’s temperate systems, if other requirements, such as oxygen levels and salinity are met. This is likely considering *A. senhousia* is also tolerant of a wide range of salinity (multiple Solent sites have reduced or fluctuating salinities) and oxygen levels^21^. Colder months in the Solent, when the average temperature is < 15 °C (e.g. winter and spring)^28^, probably limit reproduction^59^. Despite the evidence indicating a summer spawning population in the Solent, there are inconsistencies in the temperature range reportedly required for *A. senhousia* reproduction to take place. For example, a temperature of 22.5–28 °C is well documented^5,19,60^. It is possible that this temperature range only applies to *A. senhousia* individuals originating from the warmer parts of its native region^61^. A lineage that is predisposed to colder waters and has high levels of polymorphism may be responsible for adaptation to the relatively cold waters of Northern Europe^61^. Research should, therefore, focus on identifying the lineage present in this area and determining the temperature limits for reproduction. In addition, the possibility of multiple and prolonged spawning events in the UK cannot be excluded since we observed high inter-individual variability of GSI data. This is not an unusual phenomenon, with prolonged spawning (more than two months) reported outside of its native range^1,10,24,55,62^.

This study highlights that *A. senhousia* survives in multiple habitat types present in the Solent confirming the species’ capability for colonising diverse intertidal and subtidal habitats^10,34,40,51^. Due to the opportunistic collection methods for data used in this study, it is not currently possible to determine the geographical extent of the population or the rate of spread within the Solent since its arrival. Indeed, although the largest density (290 m^−2^) and greatest number of positive sites (35%) were reported from sampling of Southampton Water in 2016, there was no significant difference in median density between years. Currently, distributions in both Southampton Water and Brownwich beach appear patchy and spatially variable with lower densities than other invaded locations^10,40^. This may be in part due to limited sampling, but the *A. senhousia* populations in the Solent could be experiencing an extended lag phase which is typical of newly introduced species^63^. However, this does not necessarily mean densities will inevitably increase in the future. Local factors might prevent mat formation, for example, anoxia associated with warmer months can induce mass mortalities^23,64,65^. *Arcuatula senhousia* is also predated upon by shorebirds birds (diving ducks and oyster catchers)^8,62,66^, boring carnivorous gastropods^51,67,68^, fish^15^ and probably crustaceans and echinoderms due to its thin shell. Therefore, intense activity by predators may limit *A. senhousia*’s mat-forming abilities. In conclusion, further data to describe the distribution of *A. senhousia*’s in the Solent are required.

### Potential effects on European natural capital

Non-native species impact natural capital and thus alter the value of ecosystems in terms of the ecosystem goods and services provided. Tables 2, 3, 4 and 5 provide summaries of potential impacts (both positive and negative) associated with *A. senhousia* on ecosystem services (addressing the categories of Provisioning, Regulating, Supporting and Cultural) and identifies key knowledge gaps which should be addressed in the short term as a priority.

**Table 2:** A summary of the impacts of *A. senhousia* in relation to Provisioning ecosystem services. ( +) denotes a potentially positive impact, (–) denotes a potentially negative impact. Priority questions are those that should be addressed by researchers to generate a full risk assessment and management plan.

Provisioning ecosystem services	+ /−	*A. senhousia* impacts and observations	+ /−	Supporting information	+ /−	Priority questions
Food (wild, farmed)	–	Biofouling organism. Attached to *O.edulis* and concrete plates (this study); Hong Kong oyster (*Crassostrea hongkongensis*)^37^; synthetic capron line (126,000 spat/m^2^)^3^	–	Spawning may overlap with *Mytilus* spp. in Europe: *A. senhousia* spawning prolonged in introduced range^1,19,24,55,62,96^; gonad ripening by July in UK (this study); documented hybridisation amongst *Mytilus* spp.^90,96–98^	–	Disrupts the cultivation of commercial species through biofouling (i.e. more intense cleaning required)?
					–	Disrupts the cultivation of commercial species through resource competition?
					–	Introduces diseases which impact commercial species?
	–	Reduces clam (*Chione* spp., *Mactra* spp., *Meretrix lusoria*, *Ruditapes philippinarum*) growth and survivorship via space and food competition and by increasing predation^69–72^	–	Introduced bivalve molluscs can facilitate the spread of shellfish diseases^89^	−	Hybridises with commercial and native species, influencing genetic diversity?
					+ /−	Consumed by people in introduced range?
	+	Human consumption in China^22,95^				
Animal feed (wild, farmed, bait)	+	Fish bait and feed stock for shrimp and crab aquaculture in Japan^21^	+	Mollusc shells used as poultry grit^94,99^	+	Use as poultry grit?
Pet trade products		–	+	Mollusc shells used for pet bird nutrition and aquarium pH buffer^94,99^	+	Use as pet bird nutrition and aquarium pH buffer?
Fertilizer		–	+	Mollusc shells used as soil conditioner^94,99^	+	Use as soil conditioner?
Aggregates extraction		–	+	Mollusc shells are used for: construction materials; biofilter medium; calcium acetate road de–icer^94,99^	+	Use as: construction materials; biofilter medium; calcium acetate road de–icer?

**Table 3:** A summary of the impacts of *A. senhousia* in relation to Regulating ecosystem services. ( +) denotes a potentially positive impact, (–) denotes a potentially negative impact. Priority questions are those that should be addressed by researchers to generate a full risk assessment and management plan.

Regulating ecosystem services	+ /−	*A. senhousia* impacts and observations	+ /−	Supporting information	+ /−	Priority questions
Waste (excess nutrients, toxic pollutants) remediation	+	Removes excess nitrogen and phosphorus from water^100^ (excess nutrients are detrimental to *Zostera* spp)^84^	+	Mussels such as *M. edulis* sequester and store toxic pollutants (mutagenic/ carcinogenic hydrocarbons, heavy metals, micro plastics, nanoparticles, pharmaceuticals)^101^	+	Nutrient remediation (nitrogen and phosphorus): reduction in size/frequency of eutrophication events and harmful algal blooms (HABs)?
					+	Reduction of toxic pollutants in pelagic zone?
Natural hazard protection	+	*Arcuatula senhousia* mats can stabilise soft sediments^40^ likely reducing resuspension events^102^	+	Mussel mats offer protection of ecologically sensitive habitats such as seagrass beds and salt marshes by reducing shoreline and bed erosion^99^	+	Mats work as coastal sea defences?
					+	Mats reduce resuspension events?
Climate regulation	–	An additional source of CO_2_ in seawater, increasing CO_2_ evasion from seawater into the atmosphere^103^	+ /−	Bivalves can influence the carbon budget via calcification: sequestration of carbon in the form of calcium carbonate and the release of carbon in the form of CO_2_^99,104^	+ /−	Carbon source or sink?

**Table 4:** A summary of the impacts of *A. senhousia* in relation to Supporting ecosystem services. ( +) denotes a potentially positive impact, (–) denotes a potentially negative impact. Priority questions are those that should be addressed by researchers to generate a full risk assessment and management plan.

Supporting ecosystem services		*A. senhousia* impacts and observations		Supporting information		Priority questions
Provision of habitat	–	Attached to native European flat oyster (*O. edulis*) shells and roof tiles used for its cultivation where there are efforts to restore *O. edulis* populations (this study)	–	Introduction of commercial bivalve molluscs such as *Magallana gigas* can introduce non–native epifauna that hitch–hike on shells^50^	–	Interferes with native shellfish (e.g. *O. edulis*) restoration?
					** + **/−	Inhibits or facilitates seagrass (e.g. *Zostera* spp.) beds?
	** + **/−	Inhibitive and potentially facilitative effects on seagrass (*Zostera marina*)^34,36,67^*.*	** + **	Mussels such as *M. edulis* facilitate removal of fine sediment from the pelagic zone^41,108^. Likely true for *A. senohousia* since levels of fine sediment are higher within mats^40^	** + **/−	Outcompetes other invasive species?
					–	Introduces non–native shell epifauna?
	** + **/−	Directly settle on *Zostera* blades as juveniles^3^ – probably later become dislodged^105,106^. Found within beds of *Z. marina* and *Z. noltei* (this study) Causes changes in macrobenthos species community^23,40,62,107^			–	Creates habitat for other invasive species?
					** + **	Reduces smothering of benthic fauna by fine sediment?
Provision of food	** + **/−	Food source for predators: birds (diving ducks and oyster catchers)^8,62,66^, boring gastropods^51,67,68^, fish^109^ and probably crustaceans and echinoderms due to its thin shell		–	** + **/−	Causes changes in dispersal patterns and/or numbers of predators
Genetic diversity		–	–	Potential for hybridisation with native species (see row 1.2.)	−	Hybridises with commercial and native species, influencing genetic diversity?

**Table 5:** A summary of the impacts of *A. senhousia* in relation to Cultural ecosystem services. ( +) denotes a potentially positive impact, (–) denotes a potentially negative impact. Priority questions are those that should be addressed by researchers to generate a full risk assessment and management plan.

Cultural ecosystem services	+ /−	*A. senhousia* impacts and observations	+ /−	Supporting information	+ /−	Priority questions
Recreation	–	Provides habitat and food for a toxic sea slug (*Pleurobranchaea* *maculata*) in New Zealand which is harmful to dogs^110^	+ /−	Introduced molluscs such as *M. gigas* can provide feeding grounds for some shorebird spp. but destroy it for others^111^	+ /−	Impacts bird watching?
					+	Reduces *Escherichia coli* in bathing waters?
	–	Biofouling organism. Found attached to boat hulls^51^	+	Mussels such as *M. edulis* sequester and store toxic pollutants^101^	–	Increases time and money spent on cleaning boat hulls?
Visual amenity	+ /−	Forms large mats on soft sediment and can attach to hard surfaces^1,34,100,112^ (this study)		–	+ /−	Changes aesthetics of marine infrastructure and beaches?
Human health		–	–	Human enteric viruses are carried by cultured and wild mussels^113^	–	Carries bacteria or viruses harmful to humans?

#### Provisioning services

*Arcuatula senhousia* has been reported to reduce the growth rate and survivorship of commercially important clams by competing for space and food^69–71^ and indirectly increasing predation^72^. In the Solent, oysters (*O. edulis*); clams; cockles and polychaetes for angling bait are commercially harvested from intertidal and subtidal soft sediment habitats and many are important fisheries across Europe^42,73,74^. *Arcuatula senhousia* collected from Brownwich were often attached to dead *C. edule* shells, although whether this attachment resulted in the death of *C. edule* cannot be concluded. High *A. senhousia* densities can alter sediment conditions^40,41^, which may have significant implications for the macrofaunal community generally and these commercial species. In Sacca di Goro, Italy, shellfish farmers reported reduced numbers of *Ruditapes philippinarum* under *A. senhousia* mats^23^. However, *R. philippinarum* has escaped cultivation in Europe and formed invasive populations; this context should be considered when undertaking impact assessments^75,76^.

We only found one individual from Portsmouth Harbour growing within a bed of *Zostera* spp*.*, although *A. senhousia* co-occurs with seagrasses in both its native and introduced ranges^3,24,34,77,78^*.* Seagrass beds are biodiverse ecosystems providing a variety of ecosystem services across the world, such as carbon capture, coastal defence and the provision of nursery habitat for juvenile fish, including those of significant commercial value in Europe^79–83^. Since the late 1800s, seagrass beds have suffered from substantial degradation due to a host of biotic and abiotic factors (although some recent recovery has been reported)^84,85^*.* These degraded beds, and new beds transplanted for restoration schemes (for example, Project Seagrass^86^), may be at risk, since *A. senhousia* mats have been found to inhibit rhizome growth in recovering populations with low plant density (in contrast, impacts of *A. senhousia* on established beds have been reported as small and non-consistent)^34^. Solent densities (290 m^−2^) may be currently too low to impact seagrass, compared to 15,000 m^−2^ in San Diego Bay – the site of the aforementioned seagrass study^34^. Whether higher densities form in the future will depend on a complex interplay of environmental conditions and biological factors.

Within this study we found evidence for *A. senhousia* attachment to empty *O. edulis* shells, *M. edulis* shells and concrete tiles in the Hamble estuary. In a different study, *A. senhousia* was also found attached to cultured *Crassostrea hongkongensis* in Hong Kong^37^. The colonisation of locations in both fully saline and brackish European waters by *A. senhousia* could increase the cost of shellfish aquaculture via biofouling and directly compete with the commercial species for substrate and food. Biofouling has been estimated to be 20–30% of shellfish production costs, though this cost varies depending on the commercial species and the geographic location of the operation^87,88^. Disease introduction and hybridisation with commercial species are also possible outcomes that could have significant risks for the European aquaculture industry. For example, the cultivation of the non-native Pacific oyster (*Magallana gigas*) in France since 1966 is likely to have contributed to the arrival and spread of gill disease to Portuguese oysters (*Crassostrea angulata*)^89^. Further, expanding populations of *Mytilus trossulus* in the UK, likely driven by commercial mussel growing activity, have been associated with the appearance of *M. trossulus* x *M. edulis* hybrids which are less valuable as a commercial species^90^. However, at the time of writing, investigations into potential disease spread from *A. senhousia* to other shellfish, or hybridisation between *A. senhousia* and other mussels could not be found*.* Nonetheless, *A. senhousia* may be a suitable host of a native generalist parasite, the pea crab *Pinnotheres pisum,* in the UK, considering that *Pinnotheres novaezelandiae* was found within *A. senhousia* in New Zealand^91^. *Pinnotheres* spp. are known to negatively impact the condition index, oxygen consumption and filtration rate of *Mytilus* spp.^92,93^.

Any non-native species is likely to have positive and negative effects on provisioning services and this is the case for *A. senhousia*. For example, it can be eaten by humans for food^22^ or could be used to provide products to the pet trade^94^. Reusch and Williams^34^ also found it could be beneficial to seagrasses by providing nutrients and could even protect vulnerable habitats from erosion if it forms mats. Increases in habitat diversity through an increase in structural complexity from mats or aggregations of *A. senhousia* may provide significant benefits for other species and biodiversity more generally. Thus, any risk assessment needs to cover both potential negative and positive impacts so that informed management decisions can be made.

#### Regulating, supporting and cultural services

The densities currently reported are unlikely to have an influence on key regulating and supporting services at anything, but the very local scale. Nevertheless, the potential for nutrient bioremediation, carbon sequestration, water clarity improvements and habitat provision will grow if densities increase in combination with the spatial extent of the Solent’s populations across the multiple habitats. The effects on cultural services, such as human health and recreation, are some of the most difficult to predict, but could have the most direct and widespread impact on people within the region and as well as the blue economy.

Impact assessments and management plans for newly arrived species must be balanced by considering both negative *and* positive impacts, such as those in Tables 2, 3, 4 and 5, and accounting for shifting baselines (see discussion by Crooks^71^). The imperative is to answer the key questions we have posed in Tables 2, 3, 4 and 5 about the effects (both positive and negative) and the subsequent risks to European habitats and coastal economies. This requires investment in monitoring, but also examination of the potential interactions between *A. senhousia* and key habitats and species. This two-pronged approach is essential for determining whether *A. senhousia* or other biotic and abiotic factors are responsible for ecosystem change^71^. Moreover, as previous invasion trajectories of *A. senhousia* are diverse, predicting the impacts on services (and any restoration efforts to improve colonised but protected habitats) will be challenging without context-relevant experimental data. For example, Mastrototaro et al.^10^ found that a population in the Mediterranean had increased to densities of up to 3800 m^−2^ within two years of arriving. In contrast, the density of a population in Auckland, New Zealand declined by 60% in one year, decreasing from 16,000 m^−2^ to 5,500 m^2^^62^. Large temporal variation in density is typical of an opportunistic species, with highly erratic population dynamics, increasing the risk of population extinctions as well as expansions^1,23,63^. The risk of rapid non-native species population expansion emphasises the need for prompt responses to new introductions. The delay between the earliest detection of *A. senhousia*in the UK (2011) and the first published report of its arrival (2017) suggests the need for improvement of the national invasive species reporting and response systems. Furthermore, there is a need to prioritise the identified impacts of *A. senhousia* so that management resources can be effectively allocated. This requires identification of the ecosystem service/s at risk (this study), assessment of the magnitude and scale of ecosystem service impacts, and ecosystem service valuation (ESV)^114^. ESV can be done in a variety of ways including the assignment of an economic monetary value (e.g. ^115^). For impacted ecosystem services which have a direct value (such as commercial shellfish stocks) ESV is relatively simple, but for others with an indirect value, such as bioremediation, the replacement cost valuation method can be used (e.g. ^116,117^). ESV methods are still very much open to discussion^118^.

## Conclusion

Our study confirms that *A. senhousia* has been in the Solent for at least eight years, indicating stable, self-sustaining populations located on the periphery of the Greater North Sea ecoregion (and by extension Europe). We believe that *A. senhousia* is likely to spread further within this region. In fact, *A. senhousia* has already been reported from the Netherlands (in 2018), although it is not clear whether the 30 individuals collected represent an established population^119^. Where *A. senhousia* populations establish in the future will be dependent on a wide variety of factors, such as its genetic variation and phenotypic plasticity^120,121^, hydrodynamics^122^, propagule processes, and environmental conditions^123^. If the lineage in the Solent is one that is predisposed to colder water adaptation (Asif and Krug^61^ suggested this as a reason for its ability to exist in more northerly regions within its introduced range), the colonisation of diverse waters of Europe could be eminently achievable.

The presence of established, self-sustaining *A. senhousia* UK populations that can reproduce and colonise multiple habitat types, and whilst tolerating variable environmental conditions, highlights a potentially significant risk to the blue economy and natural capital within the Greater North Sea. We advocate that increased monitoring of this species is essential, especially in habitats of conservation and commercial importance. We also recommend the completion of a thorough and standardised risk assessment to aid awareness raising, inform policy and facilitate prioritisation of actions. Concurrently, determined efforts should be made to address the fundamental ecological and biological questions we have highlighted to confirm if *A. senhousia* will, soon be added to Europe’s list of *invasive* non-native species.

## Supplementary Information

Supplementary Information

## Data Availability

All raw data can be made available upon request to the authors.
