# Author Correction: Evidence for self-sustaining populations of *Arcuatula senhousia* in the UK and a review of this species’ potential impacts within Europe

**DOI:** 10.1038/s41598-021-96119-8

**Published:** 2021-08-13

**Authors:** Gordon James Watson, Jesie Dyos, Peter BarfIeld, Paul Stebbing, Kate Gabrielle Dey

**Affiliations:** 1 Institute of Marine Sciences, School of Biological Sciences, University of Portsmouth, Ferry Road, Portsmouth, PO4 9LY UK; 2 APEM Ltd, A17 Embankment Business Park, Heaton Mersey, Manchester, SK4 3GN UK

Correction to: *Scientific Reports* 10.1038/s41598-021-86876-x, published online 06 May 2021

The Supplementary Information file published with this Article contained an error in the Author list, where Hannah Tidbury was incorrectly listed as an author.

This error has now been corrected in the Supplementary Information file that accompanies the original Article.

