# Supplementary material for: Evidence for self-sustaining populations of Arcuatula senhousia in the UK and a review of this species’ potential impacts within Europe
Source: Sci Rep. 2021 May 6;11:9678. doi: 10.1038/s41598-021-86876-x (PMC8102542; doi:10.1038/s41598-021-86876-x)
Supplement: Supplementary file 1 — Supplementary Information [file 41598_2021_86876_MOESM1_ESM.docx]

Supplementary Information

**Evidence for self-sustaining populations of *Arcuatula senhousia* in the UK and a review of this species’ potential impacts within Europe**

Gordon James Watson^1^; Jesie Dyos^1^; Peter Barfield^1^; Paul Stebbing^2^; Kate Gabrielle Dey ^1*^

^1^ Institute of Marine Sciences, School of Biological Sciences, University of Portsmouth, Ferry Road, Portsmouth, PO4 9LY, UK.

^2^ APEM Ltd, International House, Southampton International, Business Park, Southampton, Hampshire, SO18 2RZ, UK.

* Corresponding author’s e-mail address: [kate.dey1@port.ac.uk](mailto:kate.dey1@port.ac.uk)

| **Location** | **Site** | **Substrate** | **First Record** | **Sampling Dates** | **Sampling Method** | **Organisation** |
| --- | --- | --- | --- | --- | --- | --- |
| Southampton Water | Intertidal | Mud/mixed sediment | 2011 | 08-09/05/2007  11-12/05/11  08-10/06/13  09-10/05/16 | Macrofauna survey; 0.1m^2^ day grab at 25 locations | Environment Agency |
| Hythe, River Test | Intertidal | Sand/mixed sediment | 2016 | 11/08/16  11/12/19 | Fish push-net survey; Riley push-net with double cod-end: 8 mm net at front end, 5 mm at cod-end (*A. senhousia* inside net)  Fish push-net survey; as above (*A. senhousia* outside of net) | Pisces Conservation Ltd |
| Brownwich | Intertidal | Mud/mixed sediment | 2017 | 25/10/17  03/09/17  15/02/18  20-21/02/19  05/05/19  19-20/05/19  03-04/07/19 | Ad hoc observations  *A. senhousia* survey; six 600 x 5 m transects | University of Portsmouth  University of Portsmouth |
| Weston Shore, River Itchen | Intertidal | Mud | 2018 | 31/03/2018 | *A. senhousia.*survey; mudflat walk | University of Southampton |
| Saxon Wharf,  River Itchen | Marina | Empty oyster shells  Concrete roof tiles, *Mytilus edulis* | 2018  2019 | 11/2018  22/02/19 | *Ostrea edulis* restoration project; cages deployed for two years  *O. edulis* settlement survey; tiles deployed for six months | University of Portsmouth |
| Port Hamble,  River Hamble | Marina | Cockle and *Ulva* sp. (caught within cage) | 2019 | 15/04/2019 | *O. edulis* restoration project; cages deployed for nine months | University of Portsmouth |
| Lepe | Intertidal | Mixed sediment with pebbles and cobbles | 2019 | 23/03/19 | Hampshire and Isle of Wight Wildlife Trust ‘Secrets of the Solent’ intertidal survey | Hampshire and Isle of Wight Wildlife Trust volunteer |
| Portsmouth  Harbour | Intertidal | Eelgrass beds  *Zostera marina* and *Z. noltei* | 2019 | 13/05/19 | Eelgrass quadrat survey | University of Portsmouth |
| Chichester  Harbour | - | Mud | 2019 | - | - | University of Portsmouth |
| Shamrock Quay, River Itchen | Marina | *O. edulis* cages | 2019 | 29/11/19 | *O. edulis* restoration project; cages deployed for two years | University of Portsmouth |
| Newtown, Isle of Wight | Subtidal | - | 2019 | - | Macrofauna survey; 0.1m^2^ day grab | University of Portsmouth |

**Table S1.** Summary of surveys and collections from a variety of habitats within the Solent region (*A. senhousia* was recorded from all surveys except the EA survey from 2007).

**Table S2.** Latitude and longitude in decimal degrees of sites in Southampton Water surveyed by the Environment Agency (EA) across years 2007, 2011, 2013 and 2016. *A. senhousia* density is given as per m^2^ (one grab per site).

| **2007** | | | | **2011** | | | | **2013** | | | | **2016** | | | |
| --- | --- | --- | --- | --- | --- | --- | --- | --- | --- | --- | --- | --- | --- | --- | --- |
| **Site** | **Lat.** | **Long.** | **Density**  **(m^-2^)** | **Site** | **Lat.** | **Long.** | **Density**  **(m^-2^)** | **Site** | **Lat.** | **Long.** | **Density**  **(m^-2^)** | **Site** | **Lat.** | **Long.** | **Density**  **(m^-2^)** |
| 1 | 50.879659 | -1.399261 | 0 | 46 | 50.904306 | -1.455931 | 0 | 71 | 50.822708 | -1.308152 | 0 | 96 | 50.914045 | -1.383556 | 0 |
| 2 | 50.887028 | -1.407057 | 0 | 47 | 50.901975 | -1.427217 | 30 | 72 | 50.828678 | -1.297458 | 0 | 97 | 50.907540 | -1.382774 | 0 |
| 3 | 50.891285 | -1.411339 | 0 | 48 | 50.891058 | -1.410901 | 70 | 73 | 50.831959 | -1.321448 | 0 | 98 | 50.893492 | -1.387623 | 40 |
| 4 | 50.901643 | -1.427406 | 0 | 49 | 50.879629 | -1.398636 | 60 | 74 | 50.832847 | -1.305801 | 0 | 99 | 50.900935 | -1.429790 | 290 |
| 5 | 50.904340 | -1.457281 | 0 | 50 | 50.902256 | -1.386997 | 0 | 75 | 50.840578 | -1.319051 | 0 | 100 | 50.892321 | -1.413615 | 0 |
| 6 | 50.908758 | -1.465792 | 0 | 51 | 50.894358 | -1.386432 | 0 | 76 | 50.843293 | -1.312862 | 0 | 101 | 50.881925 | -1.400910 | 40 |
| 7 | 50.867694 | -1.386128 | 0 | 52 | 50.905821 | -1.385968 | 10 | 77 | 50.846215 | -1.323472 | 0 | 102 | 50.873290 | -1.388457 | 10 |
| 8 | 50.894488 | -1.385506 | 0 | 53 | 50.874920 | -1.380348 | 0 | 78 | 50.854330 | -1.354254 | 0 | 103 | 50.868561 | -1.381797 | 10 |
| 9 | 50.905660 | -1.386042 | 0 | 54 | 50.913877 | -1.378864 | 0 | 79 | 50.854524 | -1.309685 | 0 | 104 | 50.862522 | -1.369046 | 0 |
| 10 | 50.908702 | -1.378165 | 0 | 55 | 50.912020 | -1.374792 | 0 | 80 | 50.863917 | -1.352956 | 0 | 105 | 50.855432 | -1.356909 | 0 |
| 11 | 50.865434 | -1.377191 | 0 | 56 | 50.868070 | -1.365774 | 0 | 81 | 50.867824 | -1.380215 | 10 | 106 | 50.850639 | -1.345483 | 0 |
| 12 | 50.861057 | -1.369165 | 0 | 57 | 50.864587 | -1.356883 | 0 | 82 | 50.868629 | -1.307473 | 0 | 107 | 50.857892 | -1.341645 | 0 |
| 13 | 50.856947 | -1.360838 | 0 | 58 | 50.858583 | -1.339689 | 0 | 83 | 50.872939 | -1.369772 | 0 | 108 | 50.864953 | -1.354789 | 0 |
| 14 | 50.851702 | -1.350739 | 0 | 59 | 50.844519 | -1.321039 | 0 | 84 | 50.875322 | -1.391784 | 0 | 109 | 50.871824 | -1.368195 | 0 |
| 15 | 50.847197 | -1.341142 | 0 | 60 | 50.862318 | -1.310749 | 0 | 85 | 50.876297 | -1.380586 | 0 | 110 | 50.874065 | -1.375058 | 0 |
| 16 | 50.832540 | -1.324039 | 0 | 61 | 50.875112 | -1.307334 | 0 | 86 | 50.879351 | -1.298814 | 0 | 111 | 50.881909 | -1.387519 | 70 |
| 17 | 50.828473 | -1.318887 | 0 | 62 | 50.878593 | -1.299863 | 0 | 87 | 50.882352 | -1.387897 | 10 | 112 | 50.832306 | -1.322452 | 30 |
| 18 | 50.823771 | -1.312964 | 0 | 63 | 50.870730 | -1.387211 | 0 | 88 | 50.883809 | -1.403672 | 70 | 113 | 50.823138 | -1.309367 | 0 |
| 19 | 50.830692 | -1.301815 | 0 | 64 | 50.863668 | -1.369869 | 0 | 89 | 50.892799 | -1.413865 | 10 | 114 | 50.828563 | -1.294790 | 0 |
| 20 | 50.834882 | -1.307688 | 0 | 65 | 50.856450 | -1.358785 | 10 | 90 | 50.894202 | -1.387543 | 0 | 115 | 50.853624 | -1.309556 | 0 |
| 21 | 50.840592 | -1.310814 | 0 | 66 | 50.847664 | -1.341065 | 0 | 91 | 50.900585 | -1.426267 | 0 | 116 | 50.878884 | -1.299020 | 10 |
| 22 | 50.847859 | -1.307823 | 0 | 67 | 50.833197 | -1.322595 | 0 | 92 | 50.903440 | -1.455215 | 0 | 117 | 50.864621 | -1.309380 | 0 |
| 23 | 50.851078 | -1.307804 | 0 | 68 | 50.840667 | -1.311310 | 0 | 93 | 50.907699 | -1.382274 | 0 | 118 | 50.839329 | -1.316229 | 0 |
| 24 | 50.863289 | -1.310650 | 0 | 69 | 50.824097 | -1.310318 | 0 | 94 | 50.910176 | -1.376225 | 0 | 119 | 50.845663 | -1.322826 | 0 |
| 25 | 50.865675 | -1.308213 | 0 | 70 | 50.829810 | -1.301629 | 0 | 95 | 50.913849 | -1.382193 | 0 | 120 | 50.868644 | -1.360479 | 10 |
| 26 | 50.873629 | -1.308948 | 0 |  |  |  |  |  |  |  |  |  |  |  |  |
| 27 | 50.875256 | -1.307361 | 0 |  |  |  |  |  |  |  |  |  |  |  |  |
| 28 | 50.878366 | -1.301075 | 0 |  |  |  |  |  |  |  |  |  |  |  |  |
| 29 | 50.845077 | -1.321116 | 0 |  |  |  |  |  |  |  |  |  |  |  |  |
| 30 | 50.848065 | -1.324567 | 0 |  |  |  |  |  |  |  |  |  |  |  |  |
| 31 | 50.854953 | -1.334015 | 0 |  |  |  |  |  |  |  |  |  |  |  |  |
| 32 | 50.858538 | -1.339689 | 0 |  |  |  |  |  |  |  |  |  |  |  |  |
| 33 | 50.863042 | -1.349160 | 0 |  |  |  |  |  |  |  |  |  |  |  |  |
| 34 | 50.865794 | -1.354024 | 0 |  |  |  |  |  |  |  |  |  |  |  |  |
| 35 | 50.868877 | -1.358657 | 0 |  |  |  |  |  |  |  |  |  |  |  |  |
| 36 | 50.872176 | -1.365036 | 0 |  |  |  |  |  |  |  |  |  |  |  |  |
| 37 | 50.875370 | -1.371914 | 0 |  |  |  |  |  |  |  |  |  |  |  |  |
| 38 | 50.877746 | -1.372436 | 0 |  |  |  |  |  |  |  |  |  |  |  |  |
| 39 | 50.879286 | -1.376055 | 0 |  |  |  |  |  |  |  |  |  |  |  |  |
| 40 | 50.902469 | -1.386382 | 0 |  |  |  |  |  |  |  |  |  |  |  |  |
| 41 | 50.910193 | -1.374404 | 0 |  |  |  |  |  |  |  |  |  |  |  |  |
| 42 | 50.911504 | -1.374145 | 0 |  |  |  |  |  |  |  |  |  |  |  |  |
| 43 | 50.912513 | -1.374359 | 0 |  |  |  |  |  |  |  |  |  |  |  |  |
| 44 | 50.913795 | -1.378666 | 0 |  |  |  |  |  |  |  |  |  |  |  |  |
| 45 | 50.914168 | -1.383000 | 0 |  |  |  |  |  |  |  |  |  |  |  |  |

**Table S3.** Latitude and longitude in decimal degrees of sites within the Solent where *A. senhousia* has been found since 2007. Site numbers refer to data points in Fig. 1 and data in Table S2. * Survey details regarding the specimen found at Chichester Harbour (2019) cannot be provided due to the commercial sensitivity of the location where it was found.

| **Location** | **Site** | **Latitude** | **Longitude** |
| --- | --- | --- | --- |
| Southampton Water | 1-120 | See Supplementary Table S2 | See Supplementary Table S2 |
| Hythe, River Test | 121 | 50.879278 | -1.406500 |
| Brownwich | 122 | 50.827780 | -1.268056 |
| Weston Shore, River Itchen | 123 | 50.886144 | -1.382559 |
| Saxon Wharf, River Itchen | 124 | 50.912690 | -1.378758 |
| Port Hamble, River Hamble | 125 | 50.861277 | -1.312887 |
| Lepe | 126 | 50.783080 | -1.356371 |
| Portsmouth Harbour | 127 | 50.837320 | -1.129653 |
| Chichester Harbour* | 128 | 50.795590 | -0.928219 |
| Shamrock Quay, River Itchen | 129 | 50.909717 | -1.380079 |
| Newtown, Isle of Wight | 130 | 50.723989 | -1.406662 |


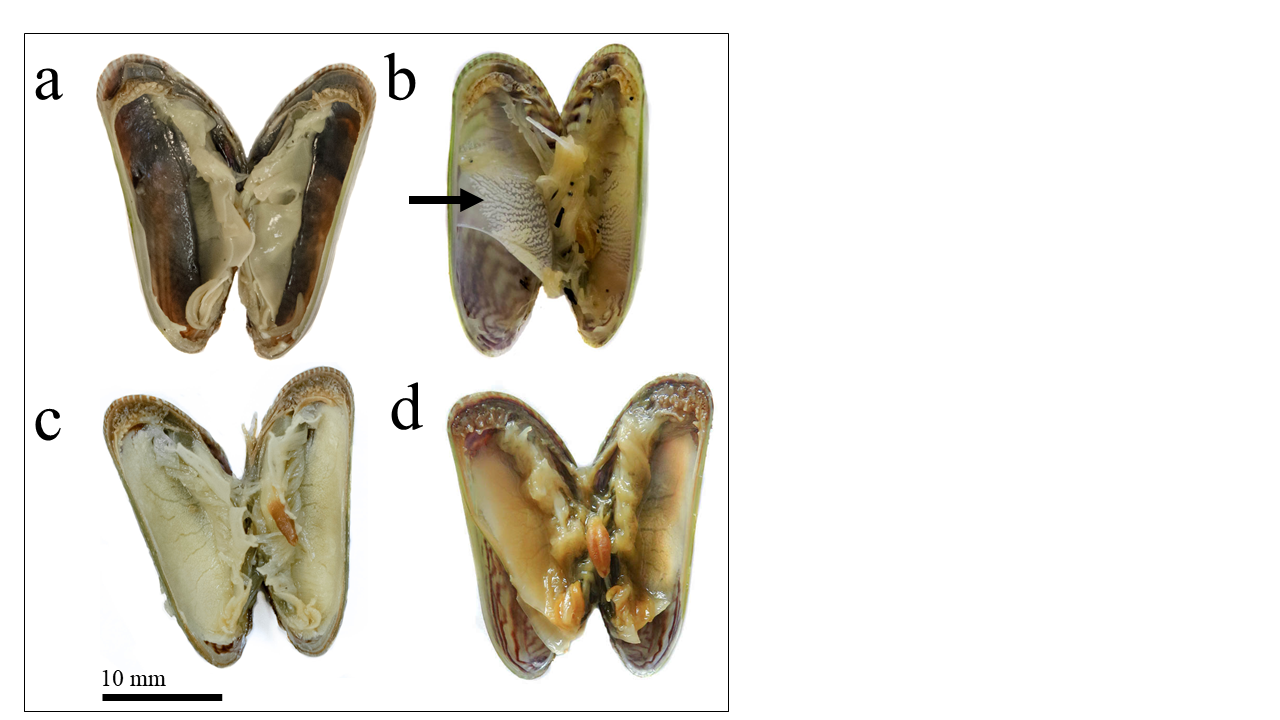


**Fig. S1** Images of gonad staging and difference in tissue colouration between *A. senhousia* males (white) (a-c) and a female (orange) (d). **a.** Gametocytes have been absorbed or gonad is spent. **b.** Gonad is spent or developing. Arrow points to translucent tissue containing follicle cells with collapsed or empty gametes. **c.** Male; gonad ripe or ready for spawning. **d.** Female; gonad ripe or ready for spawning
